# Population burden of diabetes, hypertension, and cardiovascular disease attributable to obesity: a community-based study in Türkiye

**DOI:** 10.3389/fpubh.2026.1963530

**Published:** 2026-09-29

**Authors:** Banu Ersoy Şen, Meltem Yılmaz, Merve Taştan

**Affiliations:** Faculty of Medicine, Amasya University, Amasya, Türkiye

**Keywords:** cardiometabolic diseases, cardiovascular disease, obesity, population attributable risk, prevalence

## Abstract

**Background:**

Obesity has emerged as a global public health pandemic, with rapidly increasing prevalence and a growing burden of obesity-related chronic diseases worldwide. Despite its high prevalence, obesity remains underdiagnosed, limiting effective prevention and management. Reliable, region-specific epidemiological data are therefore essential to guide public health interventions and policy development. This study aimed to examine the prevalence of obesity and its associations with chronic diseases across different age and sex groups in the community.

**Methods:**

The study included adults residing in Amasya, northern Türkiye. Data were collected at field booths between 15 June and 31 July 2025. Height and weight were measured using standardized procedures, while chronic diseases were self-reported. Associations of obesity with diabetes, hypertension, and cardiovascular disease were assessed using age- and sex-adjusted logistic regression, including interaction analyses. Attributable measures were also calculated.

**Results:**

The study included 22,667 adults (50.6% women), with a mean age of 46.4 ± 16.1 years. The prevalence of obesity was 29.9% and was higher among women than men (35.7% vs. 23.9%). The prevalences of diabetes mellitus, hypertension, and cardiovascular disease were 12.1, 16.2, and 6.6%, respectively, and were substantially higher among individuals with obesity than among those without obesity. After adjustment for age and sex, obesity was associated with higher odds of diabetes mellitus [adjusted odds ratio (aOR) = 2.88, 95% CI 2.65–3.14], hypertension (aOR = 2.50, 95% CI 2.31–2.70), and cardiovascular disease (aOR = 2.29, 95% CI 2.05–2.56). The adjusted population attributable fractions of obesity were 31.5% for diabetes mellitus, 25.8% for hypertension, and 26.1% for cardiovascular disease. Age significantly modified the associations between obesity and all three chronic conditions, whereas no significant interaction with sex was observed.

**Conclusion:**

This community-based study demonstrates that obesity is highly prevalent and strongly associated with DM, HT, and CVD. The substantial population-level burden of these conditions statistically attributable to obesity further underscores its importance as a major public health problem. These findings support strengthening evidence-based strategies that promote healthy weight through lifestyle modification and population-based prevention approaches.

## Introduction

The prevalence of obesity has increased significantly worldwide and is now considered a global pandemic. In most countries, the burden of obesity-related diseases continues to rise, posing substantial public health and socioeconomic challenges (1). According to the World Health Organization (WHO), approximately 2.5 billion adults worldwide are living with overweight or obesity, placing substantial strain on healthcare systems and negatively affecting socioeconomic development (2). Despite the high prevalence of obesity, diagnosis rates remain low. The underdiagnosis of obesity may result from multiple factors, including the prioritization of obesity-related complications over obesity itself during healthcare visits, as well as challenges related to ICD coding and reimbursement for obesity care (3).

Obesity is increasingly recognized not merely as a consequence of energy imbalance but as a chronic disease characterized by systemic low-grade inflammation and metabolic dysfunction (4, 5). Excess adipose tissue promotes chronic inflammation by secreting a wide range of proinflammatory cytokines and adipokines, which subsequently lead to insulin resistance, endothelial dysfunction, hepatic lipid accumulation, and metabolic dysregulation (5). These pathophysiological mechanisms substantially increase the risk of several chronic cardiometabolic conditions, including dyslipidemia, type 2 diabetes mellitus (DM), non-alcoholic fatty liver disease, hypertension (HT), and cardiovascular diseases (CVD) (6). Obesity-associated complications rarely occur in isolation; rather, they tend to cluster, forming complex comorbidity patterns that worsen overall clinical outcomes and complicate disease management strategies (7, 8). The rising prevalence of obesity represents a major metabolic risk factor that increases the risk of non-communicable diseases, including cardiovascular disease, cancer, chronic respiratory diseases, and diabetes (9, 10). It is estimated that obesity contributes to approximately 20% of all cancers (11). A cohort study using prospectively collected data from the Health Improvement Network database demonstrated that some individuals with obesity classified as “metabolically healthy” remain at increased risk compared with individuals with normal weight and without metabolic risk factors, including a 49% higher risk of coronary heart disease, a 7% higher risk of cerebrovascular disease, and a 96% higher risk of heart failure (12).

For these reasons, obtaining up-to-date and region-specific surveillance data on obesity and its associated diseases is of critical importance for planning effective interventions, developing health policies, and improving clinical management. In this study, we aimed to examine the prevalence of obesity and its associations with DM, HT, and CVD across different age and sex groups among adults residing in Amasya, Türkiye, thereby providing evidence to inform the development of targeted intervention strategies.

## Materials and methods

### Study design

This study was designed as a cross-sectional, community-based investigation.

### Study setting and population

The study was conducted in the city of Amasya, located in northern Türkiye, with a population of 343,900 according to the Turkish Statistical Institute (TURKSTAT), 2024. The study population consisted of adult individuals aged 18 years and older who were residing in Amasya during the study period. Participants were recruited, and data were collected between 15 June 2025 and 31 July 2025. Written informed consent was obtained from all participants prior to their participation in the study. A total of 22,667 individuals were included in the study.

### Participant selection and data collection procedure

During the data collection process, field booths were established in public areas within the city center of Amasya and the surrounding districts. To reach individuals with diverse sociodemographic characteristics, the booths were located in city and district centers, high-pedestrian-traffic public areas, and areas adjacent to healthcare facilities. Individuals who approached the booths and agreed to participate in the study were assessed according to the inclusion criteria. All participants who met the eligibility criteria were enrolled in the study.

This approach was intended to reach a large and diverse participant pool across different community settings. However, participation was voluntary and depended on individuals being present at or approaching the data collection sites. The data collection form included questions regarding age and history of chronic disease. Information on age and chronic disease status was recorded based on self-reported data provided by the participants.

### Inclusion and exclusion criteria

Individuals aged 18 years and older who were residing within the administrative boundaries of Amasya at the time of the study and who provided written informed consent were eligible for inclusion. Participants with available measurements of height and body weight and with complete data on age and the presence of DM, HT, and CVD were included in the analysis.

Individuals with missing height or body weight measurements, as well as those with incomplete data on chronic disease variables, were excluded from the study. Women who reported being pregnant were also excluded from the analyses, as pregnancy may affect body composition.

### Anthropometric measurements and body mass index

Height and body weight measurements were obtained by trained field staff in accordance with standard measurement protocols. Height was measured using a fixed stadiometer, with participants standing barefoot in an upright position. During the measurement, participants’ heads were positioned in the Frankfort horizontal plane, facing straight ahead.

Body weight was measured using regularly calibrated digital scales, with participants wearing light clothing and no shoes. Participants were instructed to distribute their weight evenly on both feet, stand still without external support, and remain in position until the weight measurement stabilized on the device.

Body mass index (BMI) was calculated as body weight in kilograms divided by height in meters squared (kg/m^2^). BMI categories were defined according to the WHO classification as underweight (<18.5 kg/m^2^), normal weight (18.5–24.9 kg/m^2^), overweight (25.0–29.9 kg/m^2^), and obesity (≥30.0 kg/m^2^). For the analyses examining the association between obesity and chronic diseases, participants were classified as individuals with obesity (BMI ≥ 30.0 kg/m^2^) and individuals without obesity (BMI < 30.0 kg/m^2^).

### Definition of covariates

The presence of DM and HT was defined based on participants’ self-report of a prior physician diagnosis or current use of physician-prescribed medication specifically for the respective condition. Participants were asked whether they were currently using medication prescribed by a physician for diabetes or hypertension; individual drug names and pharmacological classes were not recorded. DM was recorded as a single disease category, and diabetes subtypes (type 1 and type 2) were not distinguished in the data collection form. The presence of CVD was defined for individuals who reported a previous physician diagnosis of coronary artery disease, myocardial infarction, stroke, or heart failure. All chronic disease variables were coded as binary variables (yes/no).

### Ethics approval

The study was conducted in accordance with the principles of the Declaration of Helsinki. Ethical approval was obtained from the Non-Interventional Clinical Research Ethics Committee of Amasya University (decision dated 12 June 2025, No. 2025/119). The necessary permissions were also obtained from the Provincial Directorate of Health.

Written informed consent was obtained from all participants prior to enrollment. Participant data were anonymized in accordance with confidentiality principles and used exclusively for scientific purposes.

### Statistical analysis

Descriptive statistics were summarized as mean, median, standard deviation, and interquartile range (first and third quartiles) for continuous variables, and as frequencies and percentages for categorical variables. The distributions of continuous variables were evaluated using histograms, normal Q–Q plots, and skewness and kurtosis values. The Kolmogorov–Smirnov test was also examined; however, given the large sample size, graphical and descriptive assessments were primarily considered when evaluating distributional characteristics. Pearson’s chi-square test was used to compare categorical variables, while the independent samples t-test was applied for normally distributed continuous variables.

The prevalence of DM, HT, and CVD was calculated according to obesity status. Prevalence estimates were determined separately for individuals with obesity (BMI ≥ 30.0 kg/m^2^) and individuals without obesity (BMI < 30.0 kg/m^2^) and were expressed as percentages.

For each outcome, relative risk (RR) was calculated as the ratio of disease prevalence among individuals with obesity to that among individuals without obesity. Attributable risk (AR) was calculated as the absolute difference in disease prevalence between the two groups. Attributable risk percentage (AR%) was calculated as the proportion of disease prevalence among individuals with obesity statistically attributable to obesity. Crude population attributable fractions (PAFs) were calculated to estimate the population-level burden of each condition statistically attributable to obesity.

The associations between obesity and DM, HT, and CVD were evaluated using separate binary logistic regression models, with each chronic disease included as the dependent variable and obesity as the primary independent variable. All models were adjusted for age and sex. Obesity-by-age and obesity-by-sex interaction terms were additionally evaluated to assess whether the associations between obesity and each chronic disease varied according to age or sex. Adjusted odds ratios (aORs), 95% confidence intervals (CIs), and *p* values were reported.

Age- and sex-adjusted population attributable fractions were estimated using the adjusted measures of association obtained from the logistic regression models together with the prevalence of obesity.

A *p* value of less than 0.05 was considered statistically significant in all analyses.

## Results

The study population consisted of 22,667 participants residing in Amasya Province, Türkiye, including 11,462 (50.6%) women and 11,205 (49.4%) men. The mean age of the participants was 46.4 ± 16.1 years. When chronic disease variables were examined, the prevalence of DM (*p* < 0.001) and HT (*p* < 0.001) was higher among women than men, while no significant difference was observed between sexes in terms of CVD (*p* = 0.181). Both the number of chronic diseases (*p* < 0.001) and the distribution of BMI categories (*p* < 0.001) differed significantly between sexes; the prevalence of obesity (≥30 kg/m^2^) was higher among women than men (Table 1).

**Table 1:** Distribution of participants descriptive characteristics by sex.

Variable	Female *N* (%)/Mean ± SD	Male *N* (%)/Mean ± SD	Total *N* (%)/Mean ± SD	*p*
Diabetes mellitus				**<0.001**^**a**^
No	9,906 (49.6)	10,053 (50.4)	19,959 (88.9)	
Yes	1,556 (57.5)	1,152 (42.5)	2,708 (12.1)	
Hypertension				
No	9,501 (49.9)	9,531 (50.1)	19,032 (84.7)	**<0.001**^**a**^
Yes	1961 (53.9)	1,674 (46.1)	3,635 (16.2)	
Cardiovascular disease				0.181^a^
No	10,736 (50.7)	10,476 (49.3)	21,212 (94.2)	
Yes	726 (48.9)	759 (51.1)	1,485 (6.6)	
Number of chronic diseases (HT/DM/CVD)				**<0.001**^**a**^
0	8,713 (49.6)	8,849 (50.4)	17,562 (78.0)	
1	1,606 (52.6)	1,446 (47.4)	3,052 (13.6)	
2	792 (57.3)	591 (42.7)	1,383 (6.1)	
3	351 (52.4)	319 (47.6)	670 (3.0)	
BMI classification (kg/m^2^)				**<0.001**^**a**^
<18.5	131 (59.5)	89 (40.5)	220 (1.0)	
18.5–24.9	3,614 (50.6)	3,522 (49.4)	7,136 (31.5)	
25.0–29.9	3,618 (42.4)	4,907 (57.6)	8,525 (37.7)	
≥30	4,089 (60.4)	2,683 (39.6)	6,772 (29.9)	
Age (years)	46.10 ± 15.89 (Median = 45; Min–Max = 18–94; Q1–Q3 = 33–59)	46.71 ± 16.25 (Median = 46; Min–Max = 18–94; Q1–Q3 = 33–60)	46.40 ± 16.08 (Median = 46; Min–Max = 18–94; Q1–Q3 = 33–59)	**0.005**^**b**^
Height (cm)	160.48 ± 7.62 (Median = 160; Min–Max = 145–195; Q1–Q3 = 155–165)	171.65 ± 8.95 (Median = 172; Min–Max = 148–199; Q1–Q3 = 166–177)	166.00 ± 10.01 (Median = 165; Min–Max = 145–199; Q1–Q3 = 158–173)	**<0.001**^**b**^
Weight (kg)	72.01 ± 12.46 (Median = 71; Min–Max = 41–111; Q1–Q3 = 63–81)	79.39 ± 11.82 (Median = 80; Min–Max = 45–111; Q1–Q3 = 71–88)	75.66 ± 12.69 (Median = 76; Min–Max = 41–111; Q1–Q3 = 67–85)	**<0.001**^**b**^
BMI (kg/m^2^)	28.11 ± 5.39 (Median = 27.66; Min–Max = 17.19–49.55; Q1–Q3 = 23.39–31.91)	27.03 ± 4.18 (Median = 26.88; Min–Max = 17.21–43.36; Q1–Q3 = 24.22–29.76)	27.58 ± 4.86 (Median = 27.17; Min–Max = 17.19–49.55; Q1–Q3 = 24.09–30.78)	**<0.001**^**b**^

a Pearson’s chi-square test was performed.

b Independent samples *t*-test was performed.

SD, standard deviation. HT, hypertension. DM, diabetes mellitus. CVD, cardiovascular disease. BMI, body mass index. Min, minimum. Max, maximum. Q, quartile. Bold values indicate statistical significance (*p* < 0.05).

Overall obesity prevalence was 29.9%, with a prevalence of 35.7% among women and 23.9% among men. Across age groups, overall obesity prevalence was 13.7% among individuals aged 18–29 years, 19.7% among those aged 30–39 years, 29.4% among those aged 40–49 years, 40.0% among those aged 50–59 years, 43.6% among those aged 60–69 years, and 41.3% among those aged ≥70 years (Table 2).

**Table 2:** Prevalence of obesity according to age group and sex.

Age group (years)	Total *N*	Women with obesity	Men with obesity	Overall obesity
		*N* (%)	*N* (%)	*N* (%)
18–29	4,114	315 (14.8)	249 (12.5)	564 (13.7)
30–39	4,458	533 (23.8)	343 (15.5)	876 (19.7)
40–49	4,387	794 (36.5)	494 (22.3)	1,288 (29.4)
50–59	4,096	1,045 (46.7)	594 (32.0)	1,639 (40.0)
60–69	3,749	934 (50.7)	701 (36.8)	1,635 (43.6)
≥70	1,863	468 (55.4)	302 (29.7)	770 (41.3)
Total	22,667	4,089 (35.7)	2,683 (23.9)	6,772 (29.9)

Logistic regression analyses showed that obesity was significantly associated with higher odds of DM, HT, and CVD after adjustment for age and sex [DM, OR: 2.88 (95% CI: 2.65–3.14); HT, OR: 2.50 (95% CI: 2.31–2.70); CVD, OR: 2.29 (95% CI: 2.05–2.56); all *p* < 0.001]. Formal interaction analyses further demonstrated significant interactions between obesity and age with respect to the presence of DM, HT, and CVD (all *p* for interaction <0.001). In contrast, no significant interactions between obesity and sex were observed for DM (*p* for interaction = 0.162), HT (*p* for interaction = 0.939), or CVD (*p* for interaction = 0.489) (Table 3).

**Table 3:** Logistic regression analyses of the associations between obesity and diabetes mellitus, hypertension, and cardiovascular disease.

Outcome	Predictor	aOR	95% CI	*p*-value	*p* for interaction
DM	Obesity (obese vs. non-obese)	2.88	2.65–3.14	<0.001	
	Age (per year)	1.046	1.043–1.049	<0.001	
	Sex (male vs. female)	1.241	1.138–1.353	<0.001	
	Obesity × age	—	—	—	**<0.001**
	Obesity × sex	—	—	—	0.162
HT	Obesity (obese vs. non-obese)	2.50	2.31–2.70	<0.001	
	Age (per year)	1.064	1.061–1.067	<0.001	
	Sex (male vs. female)	1.102	1.019–1.193	0.015	
	Obesity × age	—	—	—	**<0.001**
	Obesity × sex	—	—	—	0.939
CVD	Obesity (obese vs. non-obese)	2.29	2.05–2.56	<0.001	
	Age (per year)	1.059	1.055–1.063	0.003	
	Sex (male vs. female)	0.846	0.757–0.946	0.003	
	Obesity × age	—	—	—	**<0.001**
	Obesity × sex	—	—	—	0.489

aOR, adjusted odds ratio; CI, confidence interval; DM, diabetes mellitus; HT, hypertension; CVD, cardiovascular disease. Reference categories were non-obese for obesity status and female for sex. Age was included as a continuous variable. *p* for interaction refers to the statistical significance of the obesity × age or obesity × sex interaction term. Bold values indicate statistical significance (*p* < 0.05).

The prevalence of DM, HT, and CVD was higher among obese individuals than among non-obese individuals. The relative risks were 3.22 for DM, 2.73 for HT, and 2.85 for CVD. The attributable risks were 15.9% for DM, 18.3% for HT, and 7.8% for CVD, corresponding to attributable risk percentages of 68.8, 63.3, and 65.0%, respectively. The crude PAFs were 39.8% for DM, 34.1% for HT, and 35.6% for CVD. After adjustment for age and sex, the PAFs were 31.5, 25.8, and 26.1%, respectively (Table 4).

**Table 4:** Prevalence of diabetes mellitus, hypertension, and cardiovascular disease by obesity status and attributable risk estimates.

Measure	Diabetes mellitus	Hypertension	Cardiovascular disease
Prevalence (non-obese)	7.2%	10.6%	4.2%
Prevalence (obese)	23.1%	28.9%	12.0%
Relative risk (RR)^a^	3.22	2.73	2.85
Attributable risk (AR)^b^	15.9%	18.3%	7.8%
Attributable risk percent (AR%)^c^	68.8%	63.3%	65.0%
Population attributable fraction (PAF, crude)^d^	39.8%	34.1%	35.6%
Adjusted ARR^a^	2.54	2.16	2.18
Adjusted PAF^d^	31.5%	25.8%	26.1%

DM, diabetes mellitus; HT, hypertension; CVD, cardiovascular disease; RR, relative risk; ARR, adjusted relative risk; AR, attributable risk; PAF, population attributable fraction.

a RR was calculated as the ratio of disease prevalence in obese individuals to that in non-obese individuals; adjusted RR was derived from the age- and sex-adjusted odds ratio.

b AR was calculated as the absolute difference in disease prevalence between obese and non-obese individuals.

c AR% represents the proportion of disease prevalence among obese individuals attributable to obesity.

d PAF was calculated using obesity prevalence and the corresponding RR; adjusted PAF was calculated using the corresponding adjusted RR.

## Discussion

This study provided an opportunity to evaluate the prevalence of obesity and obesity-attributable chronic diseases among 22,667 adult individuals living in a province of Türkiye. The findings indicate that approximately two-thirds of adults were either overweight (37.7%) or obese (29.9%), and that the prevalence of DM, HT, and CVD increased significantly with rising BMI levels. In addition, the prevalence of obesity was significantly higher among women than among men (35.7% vs. 23.9%).

The obesity prevalence observed in our study is higher than the most recent national figures reported for Türkiye in 2022 (20.2%) (13) and the global prevalence estimate (16.0%) (14). According to the OECD *Health at a Glance* report, Mexico (36.0%), the United States (33.5%), and New Zealand (34.3%) rank among the countries with the highest obesity prevalence, while Türkiye has a higher obesity rate than 23 of the 38 OECD countries (15).

The burden of obesity has been linked to a wide range of diseases and complications, including DM, HT, cardiovascular diseases, cancer, stroke, and osteoarthritis (16–19). Accordingly, the increasing prevalence of obesity leads to rising medical costs, productivity losses, and a substantial long-term disease burden, thereby remaining a public health issue of strategic importance for health systems and policy makers (16, 20). Global evidence, supported by the findings of our study, emphasizes that obesity represents a major societal health burden beyond an individual-level problem and should be considered a priority target for the prevention of chronic diseases (21).

In the present study, the prevalence of DM, HT, and CVD was reported as 12.1, 16.2, and 6.6%, respectively. While the observed DM prevalence was comparable to estimates reported at the national level in Türkiye, the prevalence of HT and CVD was lower than both national and international estimates (22, 23). This discrepancy may be attributable to the demographic profile and lifestyle characteristics of the study population, as well as to factors related to diagnostic awareness and data collection methods. Notably, hypertension was assessed based on self-reported physician diagnoses rather than direct blood pressure measurements, which may have resulted in underestimation of its true prevalence by failing to capture undiagnosed cases. Nevertheless, after adjustment for age and sex, individuals with obesity had nearly threefold higher odds of DM and more than twofold higher odds of both HT and CVD compared with individuals without obesity.

Overall, our findings suggest that obesity constitutes a significant public health problem in the study population, while the detection and diagnosis rates of certain chronic diseases may remain limited. Consistent with the existing literature, a substantial proportion of cases of HT and CVD may be undiagnosed, indicating that the reported prevalence figures may not fully reflect the true burden of these conditions in the population and that their actual prevalence may be higher than commonly assumed (24–26).

The clustering of obesity with DM, HT, and CVD on a shared pathophysiological basis provides not only statistical but also biological plausibility for these associations. The higher prevalence of these conditions among obese individuals reflects concurrent increases in dysregulated energy metabolism, insulin resistance, and vascular stress. Together with our finding that obesity remained strongly associated with all three conditions after adjustment for age and sex, these biological mechanisms support the observed relationship between obesity and cardiometabolic disease burden (27, 28).

Obesity prevalence also showed a marked age-related pattern, increasing from early adulthood and reaching its highest level in the 60–69-year age group. This age-related increase was particularly pronounced among women, with more than half of women aged 70 years or older having obesity. Consistent with this pattern, the overall prevalence of obesity, as well as DM and HT, was higher among women than among men, whereas CVD prevalence was comparable between sexes. However, despite these sex differences in prevalence, the association of obesity with DM, HT, and CVD did not differ significantly between women and men, suggesting that sex did not modify these associations. A global analysis reported that, with the exception of a few very high-income countries, the prevalence of overweight and obesity exceeded 30% among women and was higher than that among men in 15 of the 17 countries examined (21). Similarly, studies conducted across Canada, South America, Asia, the Middle East, and Africa have shown that the population attributable fraction (PAF) of obesity-related DM is higher in women than in men (29–31).

In addition, a study from China reported higher PAF values for hypertension attributable to both general and abdominal obesity among women compared with men (30). Another investigation demonstrated that pericellular fibrosis in visceral and subcutaneous adipose tissue among women was strongly associated with macrophage infiltration, with visceral adiposity in particular serving as a strong predictor of cardiometabolic risk markers (32). These findings suggest that sex-specific biological and adiposity-related characteristics may contribute to differences in the population burden of obesity-related disease. However, in our study, sex did not significantly modify the associations of obesity with DM, HT, or CVD.

After adjustment for age and sex, obesity remained independently associated with DM, HT, and CVD. Individuals with obesity had nearly threefold higher odds of DM and more than twofold higher odds of both HT and CVD compared with individuals without obesity. At the population level, approximately one-third of the burden of DM and one-quarter of the burden of HT and CVD were statistically attributable to obesity after adjustment for age and sex. In absolute terms, the largest difference in disease prevalence between individuals with and without obesity was observed for HT, followed by DM and CVD, highlighting the substantial absolute difference in HT prevalence associated with obesity in this population. Age also significantly modified these associations, indicating that their magnitude varied across age. This finding highlights the importance of considering age-specific differences when evaluating the cardiometabolic burden associated with obesity.

The burden of obesity-attributable chronic diseases represents a major public health challenge not only in Türkiye but also globally, as demonstrated by studies conducted across different countries. In a large-scale study conducted in Belgium in 2025 involving 11,611 participants, high BMI was reported to account for approximately 37,800 years lived with disability (YLD) and 56,000 years of life lost (YLL) (33). The Global Burden of Disease (GBD) 2021 study reported that approximately 52.2% of the global disability-adjusted life years (DALYs) attributable to type 2 DM were associated with high BMI (21).

Similarly, in a 15-year follow-up study by Cameron et al., the risk of DM among obese individuals increased by approximately 2.7-fold, and the proportion of DM attributable to obesity rose from 35 to 41% over time (34). With respect to HT, a study conducted by Hou et al. across 46 low- and middle-income countries reported that 38.1% of hypertension cases were attributable to obesity (35).

In an analysis covering the past 20 years in the United States, obesity-attributable HT rates were shown to have increased substantially over time, with the proportion of HT risk attributable to obesity rising from 12 to 15% among women and from 8 to 13% among men (36).

With respect to CVD, a study by Yeh et al. reported that the population attributable fraction (PAF) for CVD related to high BMI was 18.0%, with corresponding values of 19.6% in men and 15.6% in women (37). Furthermore, a meta-analysis demonstrated that overweight status accounted for approximately 45% of the risk of coronary heart disease through its adverse effects on blood pressure and cholesterol levels, while also showing that obesity independently and significantly increased the risk of coronary heart disease beyond these established risk factors (38).

Several limitations should be acknowledged. First, disease-related data were based on self-reports without biochemical confirmation or direct clinical measurements. Therefore, the possibility that some participants had undiagnosed chronic conditions cannot be excluded. Second, several important variables that may influence disease risk—such as dietary habits, smoking status, and physical activity levels—were not assessed in this study. Third, the analyses relied solely on current BMI measurements, precluding the assessment of temporality or causal relationships. Accordingly, the estimated PAFs should be interpreted as measures of the population-level burden statistically attributable to obesity rather than as the proportion of disease cases that would necessarily be prevented by eliminating obesity. Fourth, only individuals for whom height and weight measurements could be obtained were included in the study. As participation may have been influenced by individuals’ visible body weight or health perceptions, this may have resulted in the exclusion of certain groups and introduced potential selection bias. In addition, because recruitment was conducted at field booths located in public areas and near healthcare facilities, individuals with chronic diseases accompanied by substantial mobility or activity limitations may have been less likely to participate. This may have resulted in the underrepresentation of individuals with more severe chronic conditions and potential underestimation of disease prevalence. Despite these limitations, the large sample size and community-based design substantially strengthen the reliability and generalizability of the study findings.

In this context, healthcare professionals should prioritize evidence-based interventions that promote healthy dietary patterns, regular physical activity, and behavior-change–oriented health education to support individuals in maintaining healthy BMI levels.

Future research should employ longitudinal study designs to assess temporal trends in obesity and obesity-related chronic diseases and should be conducted using more comprehensive datasets that incorporate lifestyle factors, socioeconomic status, and environmental determinants. Given the observed variation in the association between obesity and chronic diseases across age, future studies should also investigate age-specific patterns and the mechanisms underlying these differences. In addition, randomized controlled trials evaluating the effectiveness of community-based intervention programs would strengthen the scientific evidence base for policies aimed at obesity prevention and control.

## Conclusion

In conclusion, this study provides region-specific data on the burden of obesity and obesity-related chronic diseases while offering insights that are also relevant at the national and global levels. Obesity affected nearly one-third of the study population and was independently associated with substantially higher odds of DM, HT, and CVD after adjustment for age and sex. Moreover, the population-level analyses indicated that a considerable proportion of the burden of these chronic conditions was statistically attributable to obesity. The observed prevalence estimates and age-related differences underscore the urgency and public health importance of addressing obesity as a priority health issue. These findings support the need for population-based obesity prevention and control strategies as part of broader efforts to reduce the burden of cardiometabolic diseases.

## Data Availability

The data underlying this study are institutional data and are not publicly available. Access may be provided subject to institutional permission.
